# 4-Chloro-*N*-(3-methoxy­phen­yl)­benz­amide

**DOI:** 10.1107/S1600536808029899

**Published:** 2008-09-20

**Authors:** Aamer Saeed, Rasheed Ahmad Khera, Naeem Abbas, Jim Simpson, Roderick G. Stanley

**Affiliations:** a Department of Chemistry, Quaid-i-Azam University, Islamabad 45320, Pakistan; b Department of Chemistry, University of Otago, PO Box 56, Dunedin, New Zealand

## Abstract

The title benzamide derivative, C_14_H_12_ClNO_2_, crystallizes with two independent mol­ecules in the asymmetric unit. Both are close to being planar, with dihedral angles between the two benzene rings of 11.92 (6) and 12.80 (7)°. In the crystal structure, N—H⋯O hydrogen bonds link mol­ecules into chains along *a*. These inter­actions are augmented by C—H⋯O hydrogen bonds to form two-dimensional layers in the *ac* plane. Additional C—H⋯O inter­actions result in a three-dimensional network consisting of undulating rows along *c*. The crystal studied was an inversion twin with a 0.59 (3):0.41 (3) domain ratio.

## Related literature

For background on the applications of benzanilides, see: Zhichkin *et al.* (2007 ▶); Igawa *et al.* (1999 ▶). For reference structural data, see: Allen *et al.* (1987 ▶).
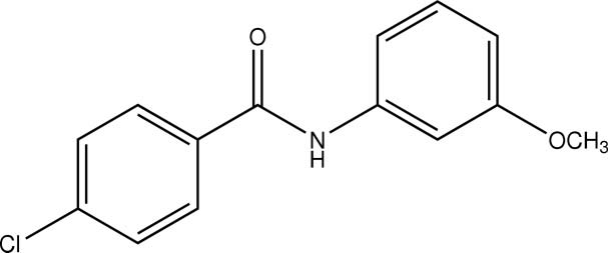


## Experimental

### 

#### Crystal data

C_14_H_12_ClNO_2_
*M* *_r_* = 261.70
Orthorhombic, 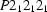

*a* = 9.6952 (4) Å
*b* = 10.5671 (3) Å
*c* = 24.3512 (8) Å
*V* = 2494.78 (15) Å^3^
*Z* = 8
Mo *K*α radiation
μ = 0.30 mm^−1^
*T* = 91 (2) K
0.80 × 0.27 × 0.18 mm

#### Data collection

Bruker APEXII CCD area-detector diffractometer
Absorption correction: multi-scan (*SADABS*; Bruker, 2006 ▶) *T*_min_ = 0.771, *T*_max_ = 0.948
47170 measured reflections
8997 independent reflections
8334 reflections with *I* > 2σ(*I*)
*R*_int_ = 0.038

#### Refinement

*R*[*F* ^2^ > 2σ(*F* ^2^)] = 0.034
*wR*(*F* ^2^) = 0.087
*S* = 1.05
8997 reflections
336 parameters
H atoms treated by a mixture of independent and constrained refinement
Δρ_max_ = 0.43 e Å^−3^
Δρ_min_ = −0.26 e Å^−3^
Absolute structure: Flack (1983 ▶), 3581 Friedel pairs
Flack parameter: 0.59 (3)

### 

Data collection: *APEX2* (Bruker, 2006 ▶); cell refinement: *APEX2* and *SAINT* (Bruker, 2006 ▶); data reduction: *SAINT*; program(s) used to solve structure: *SHELXS97* (Sheldrick, 2008 ▶); program(s) used to refine structure: *SHELXL97* (Sheldrick, 2008 ▶) and *TITAN2000* (Hunter & Simpson, 1999 ▶); molecular graphics: *ORTEP-3* (Farrugia, 1997 ▶) and *Mercury* (Macrae *et al.*, 2006 ▶); software used to prepare material for publication: *SHELXL97*, *enCIFer* (Allen *et al.*, 2004 ▶) and *PLATON* (Spek, 2003 ▶).

## Supplementary Material

Crystal structure: contains datablocks global, I. DOI: 10.1107/S1600536808029899/hb2792sup1.cif

Structure factors: contains datablocks I. DOI: 10.1107/S1600536808029899/hb2792Isup2.hkl

Additional supplementary materials:  crystallographic information; 3D view; checkCIF report

## Figures and Tables

**Table 1:** Hydrogen-bond geometry (Å, °)

*D*—H⋯*A*	*D*—H	H⋯*A*	*D*⋯*A*	*D*—H⋯*A*
N1*B*—H1N*B*⋯O1*A*	0.887 (18)	1.977 (18)	2.8638 (13)	176.4 (15)
C3*B*—H3*B*⋯O1*A*	0.95	2.44	3.0436 (14)	121
C4*B*—H4*B*⋯O2*A*	0.95	2.59	3.5134 (15)	165
N1*A*—H1N*A*⋯O1*B*^i^	0.847 (18)	1.989 (18)	2.8309 (13)	172.0 (16)
C6*A*—H6*A*⋯O2*B*^i^	0.95	2.48	3.3885 (15)	161
C7*A*—H7*A*⋯O1*B*^i^	0.95	2.57	3.1611 (14)	121

Symmetry code: (i) 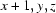.

**Fig. 1.**
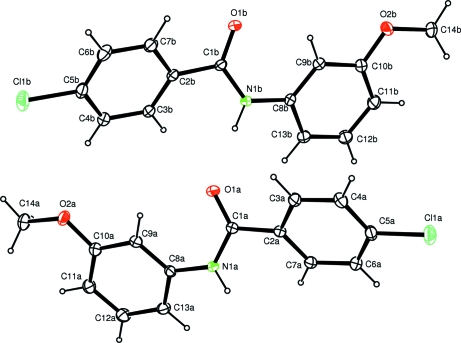
The asymmetric unit of (I) with displacement ellipsoids for the non-hydrogen atoms drawn at the 50% probability level.

**Fig. 2.**
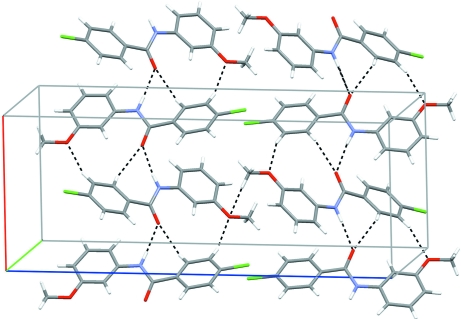
The two dimensional network in (I) formed by N—H···O and C—H···O interactions.

**Fig. 3.**
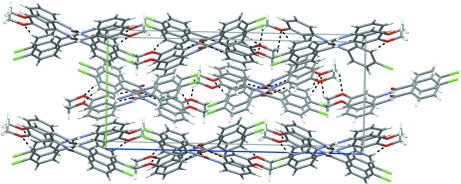
Crystal packing of (I) viewed down the a axis.

## supplementary crystallographic information

### Comment

Benzanilides have important uses in organic synthesis (e.g. Zhichkin *et al.*, 2007) and show biological activity (e.g. Igawa *et al.*, 1999).

The title compound, (I), crystallized as an inversion twin in the crystal studied with two independent molecules, A and B, in the asymmetric unit. Bond distances and angles within the molecules are normal (Allen *et al.*, 1987). Each molecule deviates slightly from planarity with dihedral angles between the two benzene rings of 11.92 (6)° for A and 12.80 (7)° for B.

In the crystal structure, N—H···O hydrogen bonds link molecules into chains along *a* (Table 1). These interactions are augmented by C—H···O hydrogen bonds to form two dimensional layers in the *ac* plane, Fig 2. Additional C—H···O interactions result in a three dimensional network consisting of undulating rows along *c*, Fig 3.

### Experimental

4-Chorobenzoyl chloride (5.4 mmol) in CHCl_3_ was treated with 3-methoxyaniline (21.6 mmol) under a nitrogen atmosphere at reflux for 4 h. Upon cooling, the reaction mixture was diluted with CHCl_3_ and washed consecutively with aqueous 1 *M* HCl and saturated aqueous NaHCO_3_. The organic layer was dried over anhydrous sodium sulfate and concentrated under reduced pressure. Crystallization of the residue from CHCl_3_ afforded the title compound (yield = 81%) as colourless needles: Analysis calculated. for C_14_H_12_Cl_N_O_2_: C 64.25, H 4.62, N 5.35%; found: C 64.19, H 4.68, N 5.30%.

### Refinement

The crystal chosen was the smallest available without having to resort to potentially damaging cutting procedures.

The N-bound H atoms were located in a difference map and refined freely with isotropic displacememt parameters. The C-bound H atoms were geometrically placed (C—H = 0.95-0.98Å) and refined as riding with *U*_iso_= 1.2*U*_eq_(C) or 1.5*U*_eq_(methyl C).  The crystal studied was an inversion twin with a 0.59 (3):0.41 (3) domain ratio.

### Crystal data

**Table:**

C_14_H_12_ClNO_2_	*F*(000) = 1088
*M**_r_* = 261.70	*D*_x_ = 1.393 Mg m^−^^3^
Orthorhombic, *P*2_1_2_1_2_1_	Mo *K*α radiation, λ = 0.71073 Å
Hall symbol: P 2ac 2ab	Cell parameters from 8842 reflections
*a* = 9.6952 (4) Å	θ = 2.3–32.7°
*b* = 10.5671 (3) Å	µ = 0.30 mm^−^^1^
*c* = 24.3512 (8) Å	*T* = 91 K
*V* = 2494.78 (15) Å^3^	Rod, colourless
*Z* = 8	0.80 × 0.27 × 0.18 mm

### Data collection

**Table:**

Bruker APEXII CCD area-detector diffractometer	8997 independent reflections
Radiation source: fine-focus sealed tube	8334 reflections with *I* > 2σ(*I*)
graphite	*R*_int_ = 0.038
ω scans	θ_max_ = 33.5°, θ_min_ = 1.7°
Absorption correction: multi-scan (SADABS; Bruker, 2006)	*h* = −14→11
*T*_min_ = 0.771, *T*_max_ = 0.948	*k* = −16→16
47170 measured reflections	*l* = −35→36

### Refinement

**Table:**

Refinement on *F*^2^	Secondary atom site location: difference Fourier map
Least-squares matrix: full	Hydrogen site location: inferred from neighbouring sites
*R*[*F*^2^ > 2σ(*F*^2^)] = 0.034	H atoms treated by a mixture of independent and constrained refinement
*wR*(*F*^2^) = 0.087	*w* = 1/[σ^2^(*F*_o_^2^) + (0.0497*P*)^2^ + 0.3361*P*] where *P* = (*F*_o_^2^ + 2*F*_c_^2^)/3
*S* = 1.05	(Δ/σ)_max_ = 0.001
8997 reflections	Δρ_max_ = 0.43 e Å^−^^3^
336 parameters	Δρ_min_ = −0.26 e Å^−^^3^
0 restraints	Absolute structure: Flack (1983), 3581 Friedel pairs
Primary atom site location: structure-invariant direct methods	Flack parameter: 0.59 (3)

### Special details

**Table:**

Geometry. All e.s.d.'s (except the e.s.d. in the dihedral angle between two l.s. planes) are estimated using the full covariance matrix. The cell e.s.d.'s are taken into account individually in the estimation of e.s.d.'s in distances, angles and torsion angles; correlations between e.s.d.'s in cell parameters are only used when they are defined by crystal symmetry. An approximate (isotropic) treatment of cell e.s.d.'s is used for estimating e.s.d.'s involving l.s. planes.
Refinement. Refinement of *F*^2^ against ALL reflections. The weighted *R*-factor *wR* and goodness of fit *S* are based on *F*^2^, conventional *R*-factors *R* are based on *F*, with *F* set to zero for negative *F*^2^. The threshold expression of *F*^2^ > σ(*F*^2^) is used only for calculating *R*-factors(gt) *etc*. and is not relevant to the choice of reflections for refinement. *R*-factors based on *F*^2^ are statistically about twice as large as those based on *F*, and *R*- factors based on ALL data will be even larger.

### Fractional atomic coordinates and isotropic or equivalent isotropic displacement parameters (Å^2^)

**Table:**

	*x*	*y*	*z*	*U*_iso_*/*U*_eq_	
C1A	0.44661 (12)	−0.01256 (11)	0.37778 (4)	0.01480 (19)	
O1A	0.33061 (9)	−0.01825 (10)	0.39873 (4)	0.0241 (2)	
C2A	0.46264 (12)	0.02718 (10)	0.31915 (4)	0.01340 (18)	
C3A	0.36632 (12)	0.11223 (11)	0.29781 (5)	0.0164 (2)	
H3A	0.2946	0.1434	0.3207	0.020*	
C4A	0.37391 (13)	0.15185 (11)	0.24357 (5)	0.0172 (2)	
H4A	0.3095	0.2113	0.2295	0.021*	
C5A	0.47765 (13)	0.10288 (10)	0.21016 (4)	0.0166 (2)	
Cl1A	0.48931 (4)	0.15338 (3)	0.142463 (11)	0.02479 (7)	
C6A	0.57279 (13)	0.01592 (11)	0.22997 (4)	0.0175 (2)	
H6A	0.6418	−0.0180	0.2065	0.021*	
C7A	0.56516 (12)	−0.02073 (10)	0.28487 (4)	0.01525 (19)	
H7A	0.6306	−0.0791	0.2991	0.018*	
N1A	0.56370 (10)	−0.04026 (9)	0.40506 (4)	0.01410 (17)	
H1NA	0.6396 (18)	−0.0282 (16)	0.3886 (7)	0.021 (4)*	
C8A	0.57503 (12)	−0.08292 (10)	0.46008 (4)	0.01315 (18)	
C9A	0.47507 (12)	−0.05885 (10)	0.49949 (4)	0.01510 (19)	
H9A	0.3933	−0.0145	0.4899	0.018*	
C10A	0.49561 (12)	−0.10044 (10)	0.55337 (4)	0.0160 (2)	
O2A	0.39068 (10)	−0.07027 (9)	0.58869 (3)	0.02047 (17)	
C14A	0.39789 (14)	−0.11940 (12)	0.64324 (5)	0.0224 (2)	
H14A	0.3922	−0.2120	0.6421	0.034*	
H14B	0.3210	−0.0859	0.6650	0.034*	
H14C	0.4854	−0.0941	0.6601	0.034*	
C11A	0.61594 (13)	−0.16314 (11)	0.56841 (5)	0.0179 (2)	
H11A	0.6298	−0.1903	0.6052	0.021*	
C12A	0.71576 (13)	−0.18529 (11)	0.52836 (5)	0.0185 (2)	
H12A	0.7989	−0.2270	0.5383	0.022*	
C13A	0.69619 (12)	−0.14772 (11)	0.47434 (5)	0.0162 (2)	
H13A	0.7641	−0.1656	0.4473	0.019*	
C1B	−0.05241 (12)	−0.00261 (10)	0.37999 (4)	0.01368 (19)	
O1B	−0.16985 (9)	−0.00419 (9)	0.36016 (3)	0.01997 (17)	
C2B	−0.02802 (12)	0.04716 (10)	0.43684 (4)	0.01360 (18)	
C3B	0.07492 (12)	−0.00094 (11)	0.47086 (4)	0.01501 (19)	
H3B	0.1339	−0.0660	0.4577	0.018*	
C4B	0.09238 (13)	0.04536 (11)	0.52393 (4)	0.0172 (2)	
H4B	0.1618	0.0118	0.5473	0.021*	
C5B	0.00613 (13)	0.14154 (10)	0.54198 (4)	0.0171 (2)	
Cl1B	0.02837 (4)	0.19996 (3)	0.608166 (12)	0.02653 (7)	
C6B	−0.09805 (13)	0.19097 (11)	0.50899 (5)	0.0193 (2)	
H6B	−0.1558	0.2570	0.5221	0.023*	
C7B	−0.11618 (13)	0.14216 (11)	0.45660 (5)	0.0174 (2)	
H7B	−0.1886	0.1732	0.4340	0.021*	
N1B	0.06041 (10)	−0.04339 (9)	0.35250 (4)	0.01450 (17)	
H1NB	0.1425 (18)	−0.0346 (16)	0.3682 (7)	0.021 (4)*	
C8B	0.06282 (12)	−0.09968 (10)	0.29958 (4)	0.01377 (19)	
C9B	−0.04236 (12)	−0.08284 (11)	0.26127 (4)	0.0157 (2)	
H9B	−0.1206	−0.0327	0.2701	0.019*	
C10B	−0.03149 (13)	−0.14050 (11)	0.20975 (4)	0.0163 (2)	
O2B	−0.14035 (10)	−0.11702 (9)	0.17528 (4)	0.02185 (18)	
C14B	−0.13097 (13)	−0.16277 (12)	0.12016 (4)	0.0205 (2)	
H14D	−0.0436	−0.1350	0.1039	0.031*	
H14E	−0.2080	−0.1292	0.0985	0.031*	
H14F	−0.1349	−0.2554	0.1202	0.031*	
C11B	0.08319 (13)	−0.21239 (11)	0.19574 (5)	0.0193 (2)	
H11B	0.0899	−0.2509	0.1606	0.023*	
C12B	0.18814 (14)	−0.22688 (12)	0.23434 (5)	0.0204 (2)	
H12B	0.2675	−0.2751	0.2251	0.024*	
C13B	0.17912 (13)	−0.17224 (11)	0.28614 (5)	0.0175 (2)	
H13B	0.2510	−0.1839	0.3122	0.021*	

### Atomic displacement parameters (Å^2^)

**Table:**

	*U*^11^	*U*^22^	*U*^33^	*U*^12^	*U*^13^	*U*^23^
C1A	0.0113 (5)	0.0201 (5)	0.0130 (4)	−0.0011 (4)	−0.0008 (4)	0.0000 (3)
O1A	0.0102 (4)	0.0468 (6)	0.0152 (4)	−0.0008 (4)	0.0008 (3)	0.0043 (4)
C2A	0.0114 (5)	0.0159 (4)	0.0129 (4)	−0.0015 (4)	−0.0004 (4)	−0.0008 (3)
C3A	0.0152 (5)	0.0199 (5)	0.0142 (4)	0.0028 (4)	−0.0003 (4)	−0.0018 (4)
C4A	0.0194 (5)	0.0167 (4)	0.0155 (4)	0.0020 (4)	−0.0035 (4)	−0.0001 (4)
C5A	0.0193 (5)	0.0188 (4)	0.0116 (4)	−0.0043 (4)	−0.0018 (4)	0.0007 (3)
Cl1A	0.03200 (17)	0.02910 (14)	0.01326 (10)	−0.00233 (12)	0.00006 (11)	0.00451 (9)
C6A	0.0160 (5)	0.0236 (5)	0.0129 (4)	0.0002 (4)	0.0015 (4)	−0.0023 (4)
C7A	0.0126 (5)	0.0188 (4)	0.0143 (4)	0.0016 (4)	−0.0010 (4)	−0.0014 (4)
N1A	0.0097 (4)	0.0202 (4)	0.0124 (4)	−0.0002 (3)	0.0005 (3)	0.0010 (3)
C8A	0.0125 (5)	0.0151 (4)	0.0118 (4)	−0.0022 (4)	−0.0016 (4)	0.0005 (3)
C9A	0.0130 (5)	0.0179 (4)	0.0144 (4)	0.0006 (4)	−0.0009 (4)	0.0008 (3)
C10A	0.0164 (5)	0.0173 (4)	0.0143 (4)	−0.0004 (4)	0.0004 (4)	0.0005 (3)
O2A	0.0190 (4)	0.0299 (4)	0.0125 (3)	0.0046 (4)	0.0033 (3)	0.0028 (3)
C14A	0.0258 (6)	0.0283 (6)	0.0129 (4)	−0.0009 (5)	0.0033 (5)	0.0034 (4)
C11A	0.0192 (5)	0.0199 (5)	0.0145 (4)	0.0013 (4)	−0.0012 (4)	0.0036 (4)
C12A	0.0170 (5)	0.0191 (5)	0.0194 (5)	0.0036 (4)	−0.0018 (4)	0.0036 (4)
C13A	0.0132 (5)	0.0182 (5)	0.0174 (5)	0.0012 (4)	0.0012 (4)	0.0019 (4)
C1B	0.0103 (5)	0.0179 (4)	0.0128 (4)	0.0003 (4)	0.0025 (3)	0.0020 (3)
O1B	0.0096 (4)	0.0351 (5)	0.0152 (3)	0.0004 (3)	0.0008 (3)	−0.0002 (3)
C2B	0.0115 (5)	0.0172 (4)	0.0121 (4)	−0.0004 (4)	0.0022 (4)	0.0015 (3)
C3B	0.0129 (5)	0.0187 (4)	0.0135 (4)	0.0023 (4)	0.0020 (4)	−0.0005 (4)
C4B	0.0157 (5)	0.0220 (5)	0.0138 (4)	0.0014 (4)	0.0010 (4)	−0.0009 (4)
C5B	0.0197 (6)	0.0184 (4)	0.0133 (4)	−0.0021 (4)	0.0040 (4)	−0.0032 (3)
Cl1B	0.03451 (17)	0.02830 (14)	0.01679 (11)	−0.00085 (13)	0.00243 (12)	−0.00894 (10)
C6B	0.0214 (6)	0.0177 (4)	0.0188 (5)	0.0048 (4)	0.0065 (4)	−0.0002 (4)
C7B	0.0160 (5)	0.0206 (5)	0.0157 (5)	0.0037 (4)	0.0030 (4)	0.0026 (4)
N1B	0.0094 (4)	0.0220 (4)	0.0121 (4)	0.0009 (3)	−0.0001 (3)	−0.0009 (3)
C8B	0.0129 (5)	0.0172 (4)	0.0113 (4)	−0.0006 (4)	0.0017 (4)	0.0005 (3)
C9B	0.0134 (5)	0.0201 (5)	0.0137 (4)	0.0022 (4)	0.0008 (4)	−0.0011 (4)
C10B	0.0158 (5)	0.0199 (5)	0.0133 (4)	0.0009 (4)	−0.0003 (4)	−0.0008 (3)
O2B	0.0181 (4)	0.0333 (5)	0.0142 (3)	0.0051 (4)	−0.0031 (3)	−0.0068 (3)
C14B	0.0219 (6)	0.0268 (5)	0.0127 (4)	−0.0008 (5)	0.0004 (4)	−0.0045 (4)
C11B	0.0203 (6)	0.0210 (5)	0.0165 (5)	0.0044 (4)	0.0011 (4)	−0.0033 (4)
C12B	0.0182 (6)	0.0234 (5)	0.0195 (5)	0.0075 (5)	0.0008 (4)	−0.0022 (4)
C13B	0.0147 (5)	0.0215 (5)	0.0164 (5)	0.0044 (4)	0.0003 (4)	−0.0004 (4)

### Geometric parameters (Å, °)

**Table:**

C1A—O1A	1.2364 (14)	C1B—O1B	1.2369 (14)
C1A—N1A	1.3475 (14)	C1B—N1B	1.3529 (14)
C1A—C2A	1.4964 (14)	C1B—C2B	1.4997 (14)
C2A—C7A	1.3932 (15)	C2B—C3B	1.3931 (15)
C2A—C3A	1.3963 (15)	C2B—C7B	1.4035 (15)
C3A—C4A	1.3875 (15)	C3B—C4B	1.3920 (15)
C3A—H3A	0.9500	C3B—H3B	0.9500
C4A—C5A	1.3933 (17)	C4B—C5B	1.3876 (16)
C4A—H4A	0.9500	C4B—H4B	0.9500
C5A—C6A	1.3884 (17)	C5B—C6B	1.3921 (17)
C5A—Cl1A	1.7364 (10)	C5B—Cl1B	1.7394 (11)
C6A—C7A	1.3939 (15)	C6B—C7B	1.3872 (16)
C6A—H6A	0.9500	C6B—H6B	0.9500
C7A—H7A	0.9500	C7B—H7B	0.9500
N1A—C8A	1.4178 (13)	N1B—C8B	1.4194 (13)
N1A—H1NA	0.847 (18)	N1B—H1NB	0.887 (18)
C8A—C9A	1.3874 (15)	C8B—C9B	1.3936 (16)
C8A—C13A	1.4033 (16)	C8B—C13B	1.4023 (16)
C9A—C10A	1.3980 (14)	C9B—C10B	1.3987 (14)
C9A—H9A	0.9500	C9B—H9B	0.9500
C10A—O2A	1.3697 (14)	C10B—O2B	1.3711 (14)
C10A—C11A	1.3908 (17)	C10B—C11B	1.3891 (17)
O2A—C14A	1.4281 (14)	O2B—C14B	1.4295 (13)
C14A—H14A	0.9800	C14B—H14D	0.9800
C14A—H14B	0.9800	C14B—H14E	0.9800
C14A—H14C	0.9800	C14B—H14F	0.9800
C11A—C12A	1.3938 (17)	C11B—C12B	1.3936 (17)
C11A—H11A	0.9500	C11B—H11B	0.9500
C12A—C13A	1.3869 (16)	C12B—C13B	1.3900 (16)
C12A—H12A	0.9500	C12B—H12B	0.9500
C13A—H13A	0.9500	C13B—H13B	0.9500
			
O1A—C1A—N1A	123.52 (10)	O1B—C1B—N1B	123.15 (10)
O1A—C1A—C2A	120.13 (10)	O1B—C1B—C2B	120.68 (10)
N1A—C1A—C2A	116.35 (10)	N1B—C1B—C2B	116.17 (10)
C7A—C2A—C3A	119.22 (10)	C3B—C2B—C7B	119.56 (10)
C7A—C2A—C1A	122.94 (10)	C3B—C2B—C1B	122.27 (10)
C3A—C2A—C1A	117.79 (10)	C7B—C2B—C1B	118.12 (10)
C4A—C3A—C2A	120.86 (10)	C4B—C3B—C2B	120.73 (10)
C4A—C3A—H3A	119.6	C4B—C3B—H3B	119.6
C2A—C3A—H3A	119.6	C2B—C3B—H3B	119.6
C3A—C4A—C5A	118.82 (11)	C5B—C4B—C3B	118.56 (11)
C3A—C4A—H4A	120.6	C5B—C4B—H4B	120.7
C5A—C4A—H4A	120.6	C3B—C4B—H4B	120.7
C6A—C5A—C4A	121.50 (10)	C4B—C5B—C6B	121.96 (10)
C6A—C5A—Cl1A	119.34 (9)	C4B—C5B—Cl1B	118.60 (9)
C4A—C5A—Cl1A	119.16 (9)	C6B—C5B—Cl1B	119.43 (9)
C5A—C6A—C7A	118.81 (10)	C7B—C6B—C5B	118.88 (10)
C5A—C6A—H6A	120.6	C7B—C6B—H6B	120.6
C7A—C6A—H6A	120.6	C5B—C6B—H6B	120.6
C2A—C7A—C6A	120.76 (10)	C6B—C7B—C2B	120.27 (11)
C2A—C7A—H7A	119.6	C6B—C7B—H7B	119.9
C6A—C7A—H7A	119.6	C2B—C7B—H7B	119.9
C1A—N1A—C8A	126.89 (10)	C1B—N1B—C8B	126.59 (10)
C1A—N1A—H1NA	117.8 (11)	C1B—N1B—H1NB	118.6 (11)
C8A—N1A—H1NA	115.3 (11)	C8B—N1B—H1NB	114.8 (11)
C9A—C8A—C13A	120.19 (10)	C9B—C8B—C13B	120.13 (10)
C9A—C8A—N1A	122.76 (10)	C9B—C8B—N1B	122.83 (10)
C13A—C8A—N1A	117.00 (10)	C13B—C8B—N1B	117.02 (10)
C8A—C9A—C10A	119.48 (10)	C8B—C9B—C10B	119.33 (10)
C8A—C9A—H9A	120.3	C8B—C9B—H9B	120.3
C10A—C9A—H9A	120.3	C10B—C9B—H9B	120.3
O2A—C10A—C11A	124.65 (10)	O2B—C10B—C11B	124.39 (10)
O2A—C10A—C9A	114.22 (10)	O2B—C10B—C9B	114.35 (10)
C11A—C10A—C9A	121.10 (10)	C11B—C10B—C9B	121.25 (11)
C10A—O2A—C14A	117.59 (9)	C10B—O2B—C14B	117.69 (9)
O2A—C14A—H14A	109.5	O2B—C14B—H14D	109.5
O2A—C14A—H14B	109.5	O2B—C14B—H14E	109.5
H14A—C14A—H14B	109.5	H14D—C14B—H14E	109.5
O2A—C14A—H14C	109.5	O2B—C14B—H14F	109.5
H14A—C14A—H14C	109.5	H14D—C14B—H14F	109.5
H14B—C14A—H14C	109.5	H14E—C14B—H14F	109.5
C10A—C11A—C12A	118.56 (10)	C10B—C11B—C12B	118.61 (10)
C10A—C11A—H11A	120.7	C10B—C11B—H11B	120.7
C12A—C11A—H11A	120.7	C12B—C11B—H11B	120.7
C13A—C12A—C11A	121.37 (11)	C13B—C12B—C11B	121.37 (11)
C13A—C12A—H12A	119.3	C13B—C12B—H12B	119.3
C11A—C12A—H12A	119.3	C11B—C12B—H12B	119.3
C12A—C13A—C8A	119.27 (10)	C12B—C13B—C8B	119.31 (11)
C12A—C13A—H13A	120.4	C12B—C13B—H13B	120.3
C8A—C13A—H13A	120.4	C8B—C13B—H13B	120.3
			
O1A—C1A—C2A—C7A	−146.54 (12)	O1B—C1B—C2B—C3B	147.65 (12)
N1A—C1A—C2A—C7A	33.66 (15)	N1B—C1B—C2B—C3B	−32.96 (15)
O1A—C1A—C2A—C3A	30.77 (16)	O1B—C1B—C2B—C7B	−29.90 (15)
N1A—C1A—C2A—C3A	−149.03 (11)	N1B—C1B—C2B—C7B	149.49 (10)
C7A—C2A—C3A—C4A	−1.76 (17)	C7B—C2B—C3B—C4B	−0.84 (17)
C1A—C2A—C3A—C4A	−179.17 (10)	C1B—C2B—C3B—C4B	−178.36 (10)
C2A—C3A—C4A—C5A	1.56 (17)	C2B—C3B—C4B—C5B	−0.79 (17)
C3A—C4A—C5A—C6A	−0.02 (17)	C3B—C4B—C5B—C6B	1.13 (17)
C3A—C4A—C5A—Cl1A	−179.20 (9)	C3B—C4B—C5B—Cl1B	−179.70 (9)
C4A—C5A—C6A—C7A	−1.29 (17)	C4B—C5B—C6B—C7B	0.20 (18)
Cl1A—C5A—C6A—C7A	177.90 (9)	Cl1B—C5B—C6B—C7B	−178.97 (9)
C3A—C2A—C7A—C6A	0.41 (16)	C5B—C6B—C7B—C2B	−1.86 (17)
C1A—C2A—C7A—C6A	177.69 (10)	C3B—C2B—C7B—C6B	2.19 (16)
C5A—C6A—C7A—C2A	1.08 (17)	C1B—C2B—C7B—C6B	179.81 (10)
O1A—C1A—N1A—C8A	1.95 (19)	O1B—C1B—N1B—C8B	−3.85 (18)
C2A—C1A—N1A—C8A	−178.25 (10)	C2B—C1B—N1B—C8B	176.77 (10)
C1A—N1A—C8A—C9A	−24.49 (17)	C1B—N1B—C8B—C9B	22.32 (17)
C1A—N1A—C8A—C13A	157.99 (11)	C1B—N1B—C8B—C13B	−159.27 (11)
C13A—C8A—C9A—C10A	−0.46 (16)	C13B—C8B—C9B—C10B	0.94 (17)
N1A—C8A—C9A—C10A	−177.90 (10)	N1B—C8B—C9B—C10B	179.31 (10)
C8A—C9A—C10A—O2A	179.44 (10)	C8B—C9B—C10B—O2B	−179.68 (10)
C8A—C9A—C10A—C11A	1.42 (17)	C8B—C9B—C10B—C11B	−1.12 (17)
C11A—C10A—O2A—C14A	−7.68 (17)	C11B—C10B—O2B—C14B	−4.22 (17)
C9A—C10A—O2A—C14A	174.37 (10)	C9B—C10B—O2B—C14B	174.29 (10)
O2A—C10A—C11A—C12A	−178.57 (11)	O2B—C10B—C11B—C12B	178.73 (12)
C9A—C10A—C11A—C12A	−0.76 (17)	C9B—C10B—C11B—C12B	0.32 (18)
C10A—C11A—C12A—C13A	−0.87 (18)	C10B—C11B—C12B—C13B	0.67 (19)
C11A—C12A—C13A—C8A	1.81 (18)	C11B—C12B—C13B—C8B	−0.83 (18)
C9A—C8A—C13A—C12A	−1.13 (17)	C9B—C8B—C13B—C12B	0.01 (17)
N1A—C8A—C13A—C12A	176.46 (10)	N1B—C8B—C13B—C12B	−178.44 (11)

### Hydrogen-bond geometry (Å, °)

**Table:**

*D*—H···*A*	*D*—H	H···*A*	*D*···*A*	*D*—H···*A*
N1B—H1NB···O1A	0.887 (18)	1.977 (18)	2.8638 (13)	176.4 (15)
C3B—H3B···O1A	0.95	2.44	3.0436 (14)	121
C4B—H4B···O2A	0.95	2.59	3.5134 (15)	165
N1A—H1NA···O1B^i^	0.847 (18)	1.989 (18)	2.8309 (13)	172.0 (16)
C6A—H6A···O2B^i^	0.95	2.48	3.3885 (15)	161
C7A—H7A···O1B^i^	0.95	2.57	3.1611 (14)	121

Symmetry codes: (i) *x*+1, *y*, *z*.
